# Modeling Stem Water Potential by Separating the Effects of Soil Water Availability and Climatic Conditions on Water Status in Grapevine (*Vitis vinifera* L.)

**DOI:** 10.3389/fpls.2019.01485

**Published:** 2019-11-22

**Authors:** Bruno Suter, Roberta Triolo, David Pernet, Zhanwu Dai, Cornelis Van Leeuwen

**Affiliations:** ^1^EGFV, Bordeaux Sciences Agro, INRA, Université de Bordeaux, ISVV, F-33882 Villenave d’Ornon, France; ^2^SOVIVINS, F-33650 Bordeaux, France

**Keywords:** grapevine, water status, stem water potential, predawn leaf water potential, maximum air temperature, modeling

## Abstract

Measuring seasonal plant water status is critical in choosing appropriate management strategies to ensure yields and quality of agricultural products, particularly in a context of climate change. Water status of grapevines is known to be a key factor for yield, grape composition, and wine quality. Predawn leaf water potential (PLWP) and stem water potential (SWP) proved to be simple and precise indicators for assessing grapevine water status and subsequent same-day spatial comparisons. A drawback of SWP is that it does not allow for temporal comparisons, because the measured value is impacted both by soil water availability and climatic conditions on the day of measurement. The objectives of this study are i) to provide a model that separates the effect of soil water content from the effect of climatic conditions on the SWP value and ii) to standardize the SWP value to a value under predefined reference climatic conditions in order to compare SWP values collected under different climatic conditions. SWP and PLWP were temporally assessed on three soil types in Saint-Émilion (Bordeaux, France) in 2015 and on five soil types in Margaux (Bordeaux, France) in 2018 using a pressure chamber. SWP measurements on two consecutive days with contrasting climatic conditions allowed to assess the impact of these conditions on SWP values. A large portion of the variability in SWP values was explained by PLWP. Model selection further showed that the addition of maximum air temperature and seasonality explained a significant amount of the remaining variability in SWP values. SWP values could be successfully standardized to a theoretical value under reference climatic conditions, which allows for temporal comparisons of SWP values. A plant-based measurement, such as the water potential, can be considered as the most straightforward indicator of plant water status as it integrates the effects of soil, plant, and atmospheric conditions. More precise interpretation of SWP values provides winegrowers with a tool to more adequately implement short- and long-term management strategies to adapt to drought in order to ensure yield and grape quality.

## Introduction

Climate change will result in an increase in temperature and an intensification of drought in many regions across the globe (IPCC, 2014). Measuring seasonal plant water status is an essential step in choosing appropriate adaptations in management strategies to ensure yield and quality of agricultural products in these changing conditions. The water status of grapevines is known to be a key factor for yield, grape composition at ripeness, and wine quality (Van Leeuwen et al., 2009).

Several techniques have been developed to measure water status in plants. Among these, predawn leaf water potential (PLWP) and stem water potential (SWP) have proven to be simple and precise indicators for assessing plant water status (Choné et al., 2001; Williams and Araujo, 2002; Shackel, 2011; Santesteban et al., 2019). However, PLWP tends to overestimate soil water availability in conditions of heterogeneous soil humidity. Under these conditions, PLWP will equilibrate with the most humid soil layer and is therefore not related to the mean water status of the soil in the root zone (Améglio et al., 1999). Another drawback of PLWP measurements is that they must be measured before dawn, which does not make it practical to implement. SWP measurements overcome this problem as they are generally measured in the early morning, at midday or in the early afternoon. SWP measured in the early afternoon is more easily implemented as there is more time available to take readings under stable conditions than at early morning when environmental conditions change more quickly (Intrigliolo and Castel, 2010). The relation between SWP and PLWP in grapevine has been shown to follow a linear function under both irrigated (Williams and Araujo, 2002; Williams and Trout, 2005) and rainfed (Zufferey and Murisier, 2007) conditions. At the same time, SWP is known to decrease with increasing vapor maximum deficit (VPD), but this relationship has been demonstrated to change over the growing season (Olivo et al., 2009) and to be different among and within species (McCutchan and Shackel, 1992; De Swaef et al., 2009; Rogiers et al., 2009; Marchin et al., 2016), and irrigation treatments (Williams and Baeza, 2007; Gálvez et al., 2014).

In order to more efficiently manage irrigation, non-stressed baselines of SWP as a function of VPD were established for prune, almond, and grapevine (McCutchan and Shackel, 1992; Williams and Baeza, 2007; Olivo et al., 2009). These baselines represent the upper boundary for steady-state water transport when soil water is readily available (Sperry et al., 2002). These baselines allow to define at which SWP values water uptake can be considered as non-limiting across a range of climatic conditions. Shackel et al. (2000) showed that this approach can reduce the use of water for irrigation while maintaining productivity in prune trees under non-limiting soil water conditions. These baselines, however, are less useful for water management in grapevines, because significant areas of grapevines are dry-farmed and a water deficit is considered favorable to wine quality, as long as it remains moderate (Van Leeuwen et al., 2009). For the same reason, even irrigated grapevines are often maintained at moderate water deficit (Matthews and Anderson, 1988). Hence, interpretation of SWP, while considering the climatic conditions at the day of measurement, is not only necessary when soil water is readily available (as in the baseline approach), but also under limiting soil water conditions. Fine-tuning the interpretation of SWP values across a wide range of water deficit would help growers in the short term to adapt to drought by means of irrigation, adjustment of the canopy size, or vineyard floor management, and in the long term by choosing appropriate plant material (rootstocks and cultivars) and planting densities (Chaves et al., 2007; Van Leeuwen and Destrac-Irvine, 2017; Van Leeuwen et al., 2019).

Grape cultivars have been classified as (near-)isohydric or (near-)anisohydric in accordance with their type of stomatal response (Chaves et al., 2010). Isohydric plants close their stomata when they sense a decrease in soil water potential or an increase in evaporative demand, while anisohydric plants allow leaf water potential to decrease with increasing VPD in order to continue gas exchange (Tardieu and Simonneau, 1998). Distinctions between the two strategies within grape cultivars, however, are often not easy to assess (Domec and Johnson, 2012). Working with previously reported (near-)anisohydry of Cabernet franc, Cabernet-Sauvignon, and Merlot (Chaves et al., 2010; Lovisolo et al., 2010; Domec and Johnson, 2012), we hypothesized the following: SWP is expected to decline with an increasingly warm and dry climate under non-limiting soil water conditions, and to become less responsive to high temperature and high VPD when soil water is limiting. Stomata close under water deficit conditions to maintain water potential above a critical minimum threshold (Hogg and Hurdle, 1997). As stomata progressively close under increasing water deficit, the impact of climatic variables on SWP values is expected to decrease.

The aims of this work are i) to provide a model that separates the effect of soil water availability from the effect of the climatic conditions on SWP value and ii) to correct the SWP value to a value under standard climatic conditions. The second objective yields a corrected SWP value that better reflects soil water availability. This value will be easier to use for strategic management decisions as it reduces the variability caused by the climatic conditions on the day of measurement.

## Materials and Methods

### Experimental Set-Up

Field experiments were carried out in 2015 (from 8 June to 9 September) in the Saint-Émilion appellation and in 2018 (from 20 June to 14 September) in the Margaux appellation in the Bordeaux area, France. Experimental plots were planted with *Vitis vinifera*(L.) cv. Merlot, Cabernet franc, and Cabernet-Sauvignon in dry-farmed commercial vineyards. Grapevines were Guyot pruned and trained with a vertical shoot positioned trellis. Plots were chosen to ensure a large range of grapevine water uptake conditions. A soil pit study allowed for the characterization of soils and grapevine-rooting profiles. In Saint-Émilion (plots M to R), soils were highly variable in texture and coarse elements content (**Table 1**). The sandy soils had a water table within the reach of the roots. At each measurement, leaves were sampled from eight adjacent grapevines. The 2015 water potential values are the average from these eight grapevines. In Margaux, plots had been selected based on historical SWP measurements from 2009 to 2017 (data not shown). The data could be distributed into three SWP thresholds, as proposed by Van Leeuwen et al. (2009). Within these thresholds three soils were chosen for the experiment. Five grapevines were selected in each plot being at least five grapevines away from the border. Measurements were always executed on the same grapevines for as long as there were enough primary leaves. Later in the 2018 season plots J, K, and L were added in order to obtain a larger range of PLWP potential values. Consequently, less measurements have been performed on these plots. The maximum distance between the experimental plots in Saint-Émilion and Margaux were 500 and 1,700 m respectively. Given the small differences in altitude and the absence of slopes, all plots were considered as located in homogeneous climatic conditions. Plant and soil properties of each plot are presented in **Table 1**.

**Table 1:** Main characteristics of the vineyards studied.

Appellation	Plot	Cultivar^1^	Rootstock	Year of planting	Grapevines per ha (spacing in m)	Row orientation	Depth (cm)	Clay (%)	Silt (%)	Sand (%)	Gravel (%)	CEC (Metson Cmol (+)/kg)	No. of measurements
Margaux	A	CS	SO4 + 3309 C	1969	10,000 (1 × 1)	NE-SW	0–100	21.9	21.5	56.6	24.5	7.89	92
Margaux	B	M	161–49 C	1959	10,000 (1 × 1)	N-S	0–125	14.3	12.7	73.0	53.1	4.52	92
Margaux	C	M	101–14 MG	1944	10,000 (1 × 1)	N-S	0–120	8.4	8.5	83.2	58.6	4.10	92
Margaux	D	CS	101–14 MG	2002	9,091 (1 × 1.1)	E-W	0–130	11.4	12.6	76.0	59.4	5.38	91
Margaux	E	CS	101–14 MG	1966	10,000 (1 × 1)	N-S	0–100	21.3	24.6	54.0	5.0	8.03	91
Margaux	F	CS	RGM	1986	10,000 (1 × 1)	N-S	0–75	33.4	47.2	19.4	8.1	10.74	92
Margaux	G	CS	NA^2^	NA	10,000 (1 × 1)	N-S	0–70	4.7	14.6	80.7	26.4	3.46	92
Margaux	H	CS	101–14 MG	1999	10,000 (1 × 1)	E-W	0–125	15.0	11.0	74.0	43.1	3.76	91
Margaux	I	M	SO4	1974	10,000 (1 × 1)	E-W	0–100	21.3	24.6	54.0	5.0	8.03	92
Margaux	J	M	SO4	1972	10,000 (1 × 1)	N-S	0–125	14.3	12.7	73.0	53.1	4.52	40
Margaux	K	M	101–14 MG	1992	10,000 (1 × 1)	E-W	0–125	15.0	11.0	74.0	43.1	3.76	50
Margaux	L	CS	101–14 MG	1999	10,000 (1 × 1)	E-W	0–60	8.1	17.1	74.8	53.6	8.37	50
Saint-Émilion	M	CF	NA	1940	6,410 (1.2 × 1.3)	N-S	0–100	42.0	30.6	27.4	5.6	14.90	16
Saint-Émilion	N	CF	RGM	1997	5,830 (1.2 × 1.4)	N-S	0–100	9.0	19.6	71.5	55.4	6.78	16
Saint-Émilion	O	CF	101–14 MG	1963	5,830 (1.2 × 1.4)	N-S	0–100	5.4	10.2	84.4	0.0	3.99	16
Saint-Émilion	P	M	RGM	1989	5,830 (1.2 × 1.4)	N-S	0–100	35.5	27.1	36.7	5.8	15.58	16
Saint-Émilion	Q	M	420A MG	2012	7,890 (1.0 × 1.3)	N-S	0–100	6.55	12.6	81.3	46.4	3.60	16
Saint-Émilion	R	M	RGM	1989	5,830 (1.2 × 1.4)	N-S	0–100	15.1	20.8	63.3	4.6	7.08	16

^1^Where CS, Cabernet-Sauvignon; M, Merlot; CF, Cabernet franc.

^2^NA, not available.

### Climatic Data

Air temperature, relative humidity, global radiation, average wind speed, and rainfall were recorded during both experiments by the nearest meteorological stations (**Table S1**). The weather stations in Saint-Émilion and Margaux were within 500 and 1,460 m from the experimental plots, respectively. The weather station of Météo-France in Saint-Émilion recorded all required data. The weather station in Margaux recorded only temperature, relative humidity, and rainfall. Global radiation data was retrieved from a weather station in Saint-Julien (distance from plots < 17.7 km) and average wind speed data from a weather station (La Crosse Technology, WS 2355, France) in Arsac (distance from plots < 5.1 km). The weather stations in Margaux and Saint-Julien were part of the CIMEL automated DEMETER network. The T_max_ data from the weather stations in Saint-Julien and Arsac correlated well with that in Margaux and followed a 1:1 line over the whole 2018 season and the sampling days in particular (r^2^ = 0.98, r^2^ = 0.94, r^2^ = 0.99, and r^2^ = 0.99, respectively). The weather stations in Saint-Julien and Arsac were located in an area with a very similar climate and grapevine phenology compared to that of Margaux (Bois et al., 2018). Reference evapotranspiration (ET_0_) was calculated according to the FAO-56 Penman-Monteith equation (Zotarelli et al., 2010). Growing degree days (GDD) were calculated with a base 0°C and summed starting from 1 April (around budbreak). The maximum VPD (VPD_max_) was calculated using the equations described by Abtew and Melesse (2013), using the variables T_max_ and minimum relative humidity.

### Water Potential Measurements

SWP values were measured with the pressure chamber (SAM Précis 2000, 33170 Gradignan, France) technique on sun-exposed and fully expanded leaves which were enclosed in an opaque plastic bag for more than 1 h to prevent transpiration and allow to reach an equilibrium with water potentials in stems (Choné et al., 2001). SWP was measured on leaves up to the sixth internode at solar noon until the early afternoon (13h30 to 17h00 local time). The operator was the same throughout the experiment to reduce human error (Levin, 2019). In this study, the PLWP measurement was considered a proxy for the soil matrix potential experienced by the roots. Despite the limitations of PLWP as reported by Améglio et al. (1999), no other easy to measure estimator to assess soil water availability exists to date for deep rooting species like grapevine. PLWP was measured prior to sunrise (between 02h00 to 06h00 local time). PLWP measurements were started no earlier than 04h00 when average PLWP was lower than −0.2 MPa. For each measurement, one leaf per grapevine was sampled. Water potentials were collected between day of year (DOY) 159–257 (from fruit set until maturity) at several occasions on two consecutive days, with SWP measurements carried out on day 1 and 2 and PLWP during the night between day 1 and 2. The underlying idea was that climatic conditions varied between day 1 and 2, while variations in soil water were minimal over two consecutive days. Hence, PLWP was considered representative for soil water availability during both days. This approach allowed creating a dataset (n=1,061) from which the effect of soil and climate on SWP values could be separated by an appropriate modeling approach.

### Statistical Analysis

Relationships between SWP, PLWP, and climatic variables were estimated via nonlinear modeling. All analyses were performed in R (R Core Team, 2017). Nonlinear models were fitted using the R function nls and models were compared using Akaike’s Information Criterion (AIC) and the Bayesian Information Criterion (BIC). The performance of each model was evaluated by the calculation of the root-mean-square error (RMSE). RMSE is the distance, on average, of a data point from a fitted line, measured along a vertical projection. A cross-validation method was applied to assess the predictive accuracy (in RMSE) of the models. A single plot was retained as the validation data for testing the models, and the remaining plots were used as training data. This procedure was repeated eighteen times (each of the eighteen plots was retained once). The assumptions of normality and equal variance were checked by quantile plots and plotting standardized model residuals against fitted values, respectively. The standardized residual plots of the models were found to be homoscedastic. Multicollinearity did not occur in any of the models (variance inflation factors < 1.2).

## Results

### Predawn Leaf Water Potential, Stem Water Potential, and Climate From June Through September in 2015 and 2018

**Figure 1:**
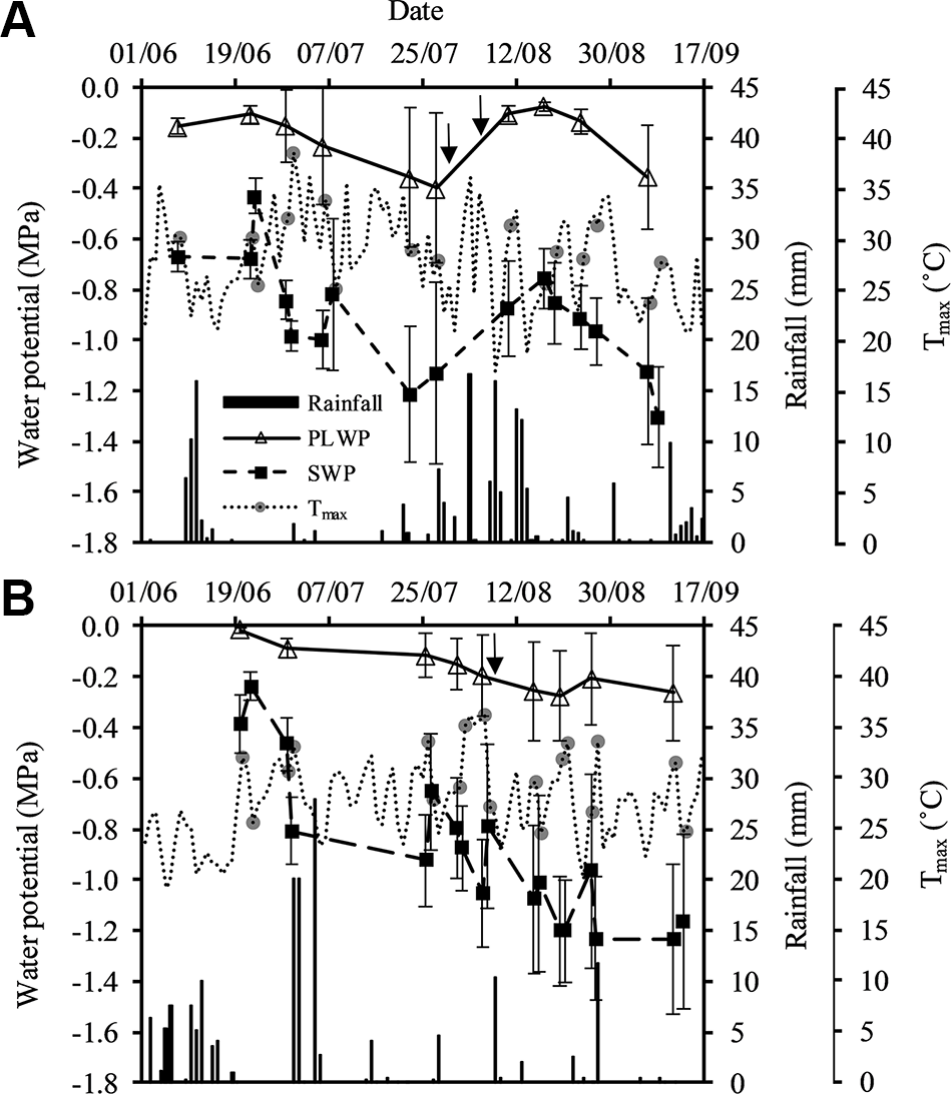
Seasonal pattern of stem water potential and predawn leaf water potential on the primary axis, and precipitation and T_max_ on the secondary axis in **(A)** Saint-Émilion in 2015 and **(B)** Margaux in 2018. T_max_ at the days of sampling are specifically represented by the gray circles. Vertical arrows represent dates of mid-veraison for Merlot (30/07) and Cabernet franc (08/08) in 2015, and Merlot and Cabernet Sauvignon (08/08) in 2018. Values are averages over plots as described in **Table 1**. Error bars indicate standard deviations.

PLWP values ranged from −0.06 to −0.86 MPa in 2015 and from −0.01 to −0.90 MPa in 2018 (**Figure 1**). SWP values ranged from −0.33 to −1.69 MPa in 2015 and from −0.12 to −1.85 MPa in 2018. Severe water deficit with SWP values below −1.4 MPa and PLWP values below −0.8 MPa were recorded in both Saint-Émilion in 2015 and in Margaux in 2018. Based on the SWP values, grapevines in Saint-Émilion experienced moderate water deficit until the end of July 2015, after which periods of precipitation replenished the soil and both SWP and PLWP recovered (**Figure 1A**). From mid-August 2015 water deficit started to increase again. In Margaux in early June 2018 all plots were at or close to field capacity and as the season progressed both SWP and PLWP decreased (**Figure 1B**). Over the course of both seasons the difference between SWP values and PLWP values generally increased. The average difference between SWP values and PLWP values at the beginning and the end of the 2015 season equaled 0.51 and 0.86 MPa, respectively. The average difference at the beginning and the end of the 2018 season equaled 0.30 and 0.94 MPa, respectively. Minimum values of PLWP and SWP for Cabernet franc, Cabernet-Sauvignon, and Merlot were, respectively, −0.61 MPa and −1.59 MPa, −0.90 MPa and −1.74 MPa, and −0.87 MPa and −1.85 MPa, respectively. SWP measured on eight leaves on five vines outside the experiment in plot A showed that SWP could on average vary by 0.18 MPa within the same grapevine (**Table S2**).

The average daily temperature from June until September in 2015 (20.7°C) and 2018 (21.6°C) were slightly higher than the 10-year average daily temperature of the Bordeaux area (20.3°C). In both seasons VPD_max_ ranged from approximately 1.2 to 5.6 kPa, and ET_0_ ranged from approximately 2 to 8 mm. Global radiation exceeded 9.74 MJ m^−2^ at all measurement days in both seasons. Total rainfall from June through September in 2015 and 2018 were very similar (176 and 165 mm, respectively), and lower than the 10-year average total rainfall in the Bordeaux area (194 mm). Using the vintage classification for drought presented in Van Leeuwen and Darriet (2016), and based on water balance modeling according to Lebon et al. (2003), the 2015 season turned out to be the second driest in Bordeaux since 1952 (after 2005), and 2018 was the ninth driest.

**Figure 2:**
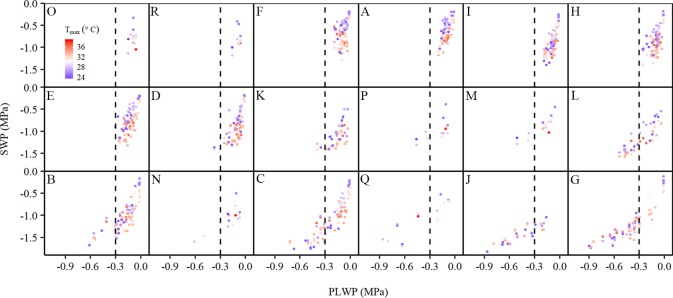
Relationship between stem water potential and predawn leaf water potential (PLWP) along a temperature gradient [T_max_, from 23.8°C (blue) to 38.9°C (red)] collected during the 2015 and 2018 seasons for each experimental plot (n=1,061). The letters correspond to the plots as specified in **Table 1**. The vertical dotted line is drawn for reference at −0.3 MPa PLWP.

Despite 2015 and 2018 being dry years in the Bordeaux area, grapevines on the experimental plots showed substantial differences in water status (**Figure 2**). In all plots SWP decreased with more negative PLWP. Plots could roughly be separated in two groups. The first group consisted of plots A, D, E, F, H, I, K, M, O, P, and R where the grapevines were rarely exposed to PLWP values lower than −0.3 MPa. The soils of plots A, E, F, I, M, P, and R contained important clay fractions (**Table 1**). Plots O and R were known to have a water table accessible to the root system. Plots M and P have a very high clay content (up to 60% in some of the soil layers) and the clay fractions are mostly composed of montmorillonite (swelling clay). It has been shown that in these soils the water is so firmly held by the clay that the grapevines have difficulties in extracting the water from the clay, which explains the occurrence of moderate to severe water deficit despite high soil water content (Tramontini et al., 2013). The second group, consisting of plots B, C, G, J, L, N, and Q, experienced moderate to severe water deficit and could be identified mostly as sandy and gravelly soils with low cation exchange capacity (**Table 1**). As described in the next section, among the climatic variables considered T_max_ explained the next largest part of the variability in SWP after PLWP. The effect of T_max_ on SWP was stronger at weak to moderate water deficit and became weaker at moderate to severe water deficit (**Figure 2**). As described in the next section, the DOY (duration through the season) was also an important factor.

**Figure 3:**
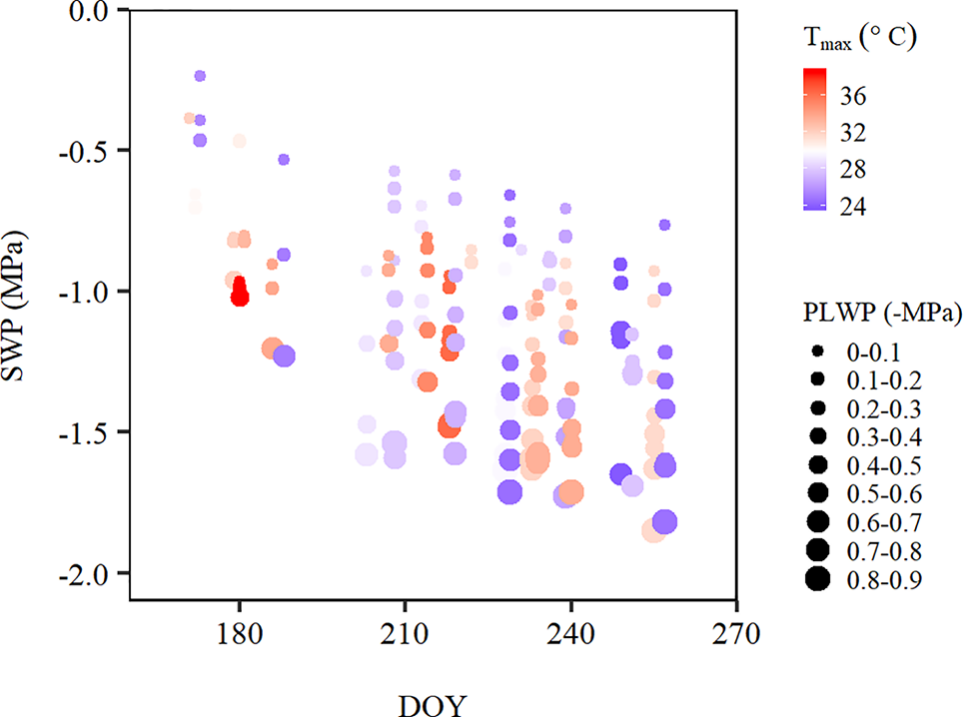
Stem water potential over the course of the 2015 and 2018 growing season along a temperature gradient [T_max_, from 23.8°C (blue) to 38.9°C (red)], where the size of the points corresponds to predawn leaf water potential (PLWP) categories in steps of 0.1 MPa. Data was averaged for each plot within each PLWP category. DOY, day of the year.

In addition to the apparent effects of PLWP and T_max_ on SWP variability, there also appeared to be a seasonal effect. For a same given PLWP and T_max_, SWP values became more negative as the season progressed (**Figure 3**).

### Modeling of Stem Water Potential

PLWP explained 66% of the variability in SWP (model 1, **Table 2**). Model selection further showed that adding climatic variables explained a significant amount of the remaining variability in SWP values (**Table 2**). Furthermore, the improvement of the predictive ability of SWP models also depended on the type of climatic variable included. The predictive ability of the climatic variables is as follows (in increasing order of AIC and BIC): T_max_ > VPD_max_. Initially, ET_0_ was considered as well but it yielded positive estimates, meaning that SWP would be more negative with lower evaporative demand. This artifact was caused by high ET_0_ recorded during the first two couples of measurements by the end of June 2018 at concurrently weakly negative PLWP and SWP. ET_0_ was shown to be strongly correlated to global radiation (r = 0.82; **Table 3**), and to decrease with DOY (r = −0.79; **Table 3**). For the climatic variables used in the models it was decided to use daily maximum values. Instantaneous T, VPD, and ΣET_0_ have also been tested, but yielded slightly higher AIC (by 4.5, 23.0, and 11.3 respectively) compared to their equivalent daily maximum values. The daily maximum versus instantaneous values of T, VPD, and ET_0_ were found to be strongly correlated (r = 0.99, 0.98, and 0.94, respectively). Hence, the use of instantaneous climatic variables is not justified, because their utilization to run the models is much more constraining and they do not improve the models. The climatic variables T_max_, VPD_max_, and ET_0_ of the measurement days were all significantly correlated (**Table 3**). The correlation among T_max_ and VPD_max_ was stronger than these variables with either ET_0_ or global radiation. The AIC of model 1 improved by 90.5 (36.3%) when adding VPD_max_ (model 2) and 139.2 (55.8%) when adding T_max_ (model 3). Modeling was therefore continued with the sole inclusion of T_max_. The model including PLWP and T_max_ (model 3) only explained part of the large variability in SWP (−0.25 down to −1.40 MPa) at PLWP between 0 and −0.25 MPa (**Figure 2**). When model 2 was fitted separately for Cabernet franc, Cabernet-Sauvignon, and Merlot, RMSE could be improved by 0.019 compared to the model containing all data, which is only a marginal gain (data not shown). This seems to indicate that the proposed models can be applied across *Vitis vinifera* varieties. Careful consideration is, however, needed since it is based on an unbalanced design and a limited number of varieties. Any of the models that contained PLWP with one of the climatic variables (VPD_max_ or T_max_) substantially improved when DOY (day of the year), i.e., seasonality, was included. PLWP correlated moderately negative with DOY (r = −0.40, P < 0.001). The inclusion of DOY to models 2 and 3 further reduced both AIC and BIC on average by 436.7 and 431.7, respectively. The coefficient for DOY in model 5 was 0.00543 (**Table 2**). This means that over the 86-day measurement period (from 20 June until 14 September) seasonality accounted for an additional 0.47 MPa decrease in SWP on top of the effect of T_max_ (**Figure 3**). In other words, for a same given T_max_ and PLWP, SWP becomes substantially more negative as the season progresses. The effect of soil moisture (PLWP), climatic conditions (T_max_), and seasonality (DOY) explained 80% of the variance in SWP (model 5). GDD were also tested in this study. When DOY was replaced by GDD in models 4 and 5, AIC was increased by 1.55 and 4.88, respectively.

**Table 2:** Comparison of goodness-of-fit and predictive power of models for SWP. Models were cross-validated by retaining one plot at a time as a validation dataset.

No.	Models	AIC	BIC	r^2^	RMSE training	RMSE cross validated
1	SWP = 1.243 · e^4.011 · PLWP^ − 1.616	−249.42	−229.55	0.659	0.214	0.216 ± 0.038
2	SWP = 1.518 · e^4.063 · PLWP^ · VPD_max_^−0.233^ − 1.614	−339.94	−315.10	0.688	0.205	0.210 ± 0.038
3	SWP = 20.164 · e^3.890 · PLWP^ · T_max_^−0.819^ − 1.628	−388.95	−363.76	0.702	0.201	0.204 ± 0.037
4	SWP = 1.479 · e^2.304 · PLWP^ · VPD_max_^−0.318^ − 0.00580 · DOY − 0.444	−792.71	−762.91	0.796	0.166	0.165 ± 0.035
5	SWP = 24.789 · e^2.144 · PLWP^ · T_max_^−0.896^ − 0.00543 · DOY − 0.579	−809.20	−779.40	0.800	0.164	0.165 ± 0.040

SWP, stem water potential; PLWP, predawn leaf water potential; VPD_max_, maximum vapor pressure deficit at the day of measurement; T_max_, maximum air temperature at the day of measurement; DOY, day of the year of measurement; AIC, Akaike’s Information Criterion; BIC, Bayesian Information Criterion; RMSE, root-mean-square error (MPa), ± standard deviation.

**Table 3:** Pearson correlation matrix of daily values of climatic variables of the measurement days (2015 and 2018).

	T_max_ (˚C)	VPD_max_ (kPa)	ET_0_ (mm)	Global radiation (MJ/m^2^)	DOY
T_max_	1				
VPD_max_	0.83	1			
ET_0_	0.40	0.63	1		
Global radiation	0.40	0.58	0.82	1	
DOY	ns*	ns	-0.79	-0.66	1

Correlations shown are highly significant (P < 0.001). *ns, not significant.

### Standardization of the Stem Water Potential Value

The nature of the dataset, with measurements performed on two consecutive days in both experimental years, allowed testing the accuracy of standardization. For this purpose, the SWP value measured on day 2 was standardized to the T_max_ on day 1, with the assumption that PLWP was maintained at the same value between day 1 and day 2. This standardized SWP (SWP_s_) could then be compared to the SWP value measured on day 1. In order to obtain SWP values under standardized climatic conditions (in this case the T_max_ of day 2, but this could be any T_max_ of interest), model 3 was algebraically rearranged. Model 3 (**Table 2**) can be rearranged as following:

(1) $$SWP_{s}=\frac{SWP+1.628}{{\left(\frac{T_{max}}{T_{s}}\right)}^{-0.819}}-1.628$$

In order to also test model 5 with the seasonality (DOY) term, only data was selected where the difference between PLWP values within a plot did not exceed 0.005 MPa and measurement days were at least a week apart. PLWP was assumed to be the same between the two data points and would thus allow for the standardization of the SWP value by T_max_ and DOY. Model 5 can be rearranged to obtain:

(2) $$SWP_{s}=\frac{SWP+0.00543\cdot DOY+0.579}{{\left(\frac{T_{max}}{T_{s}}\right)}^{-0.896}}-0.00543\cdot DOY_{s}-0.579$$

where, T_max_ represents the maximum air temperature at the day of measurement, T_s_ the temperature to which the measured SWP needs to be standardized to and DOY_s_ the DOY to which it needs to be standardized to.

**Figure 4:**
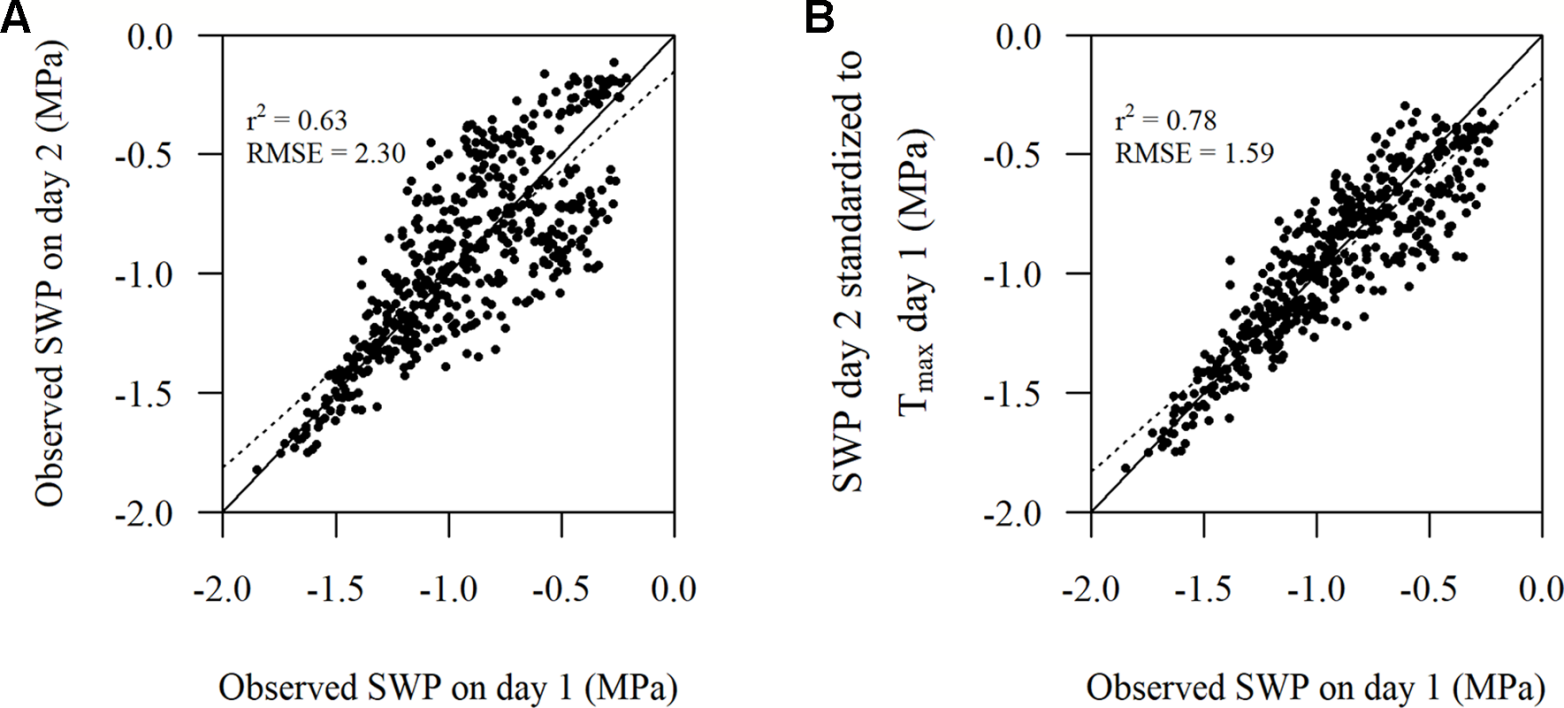
Comparison of **(A)** the observed stem water potential (SWP) on day 1 versus the observed SWP on day 2 and **(B)** the observed SWP on day 1 versus the observed SWP on day 2 standardized to T_max_ on day 1 as per Eqn. 1. The solid line represents the 1:1 line and the dotted line represents the linear regression (n = 512).

SWP values measured on day 2 standardized to T_max_ of day 1 according to Eqn. 1 improved r^2^ and reduced RMSE (**Figure 4B**) compared to the unstandardized SWP values on day 2 (**Figure 4A**). The improvement in RMSE between the observed SWP and standardized SWP by Eqn. 1 was almost 31%. When Eqn. 2 was used on the same (2-day) data the improvement was only 0.008 MPa, which was in line with the inherently low estimated coefficient (0.00543) for DOY in model 5 (**Table 2**).

**Figure 5:**
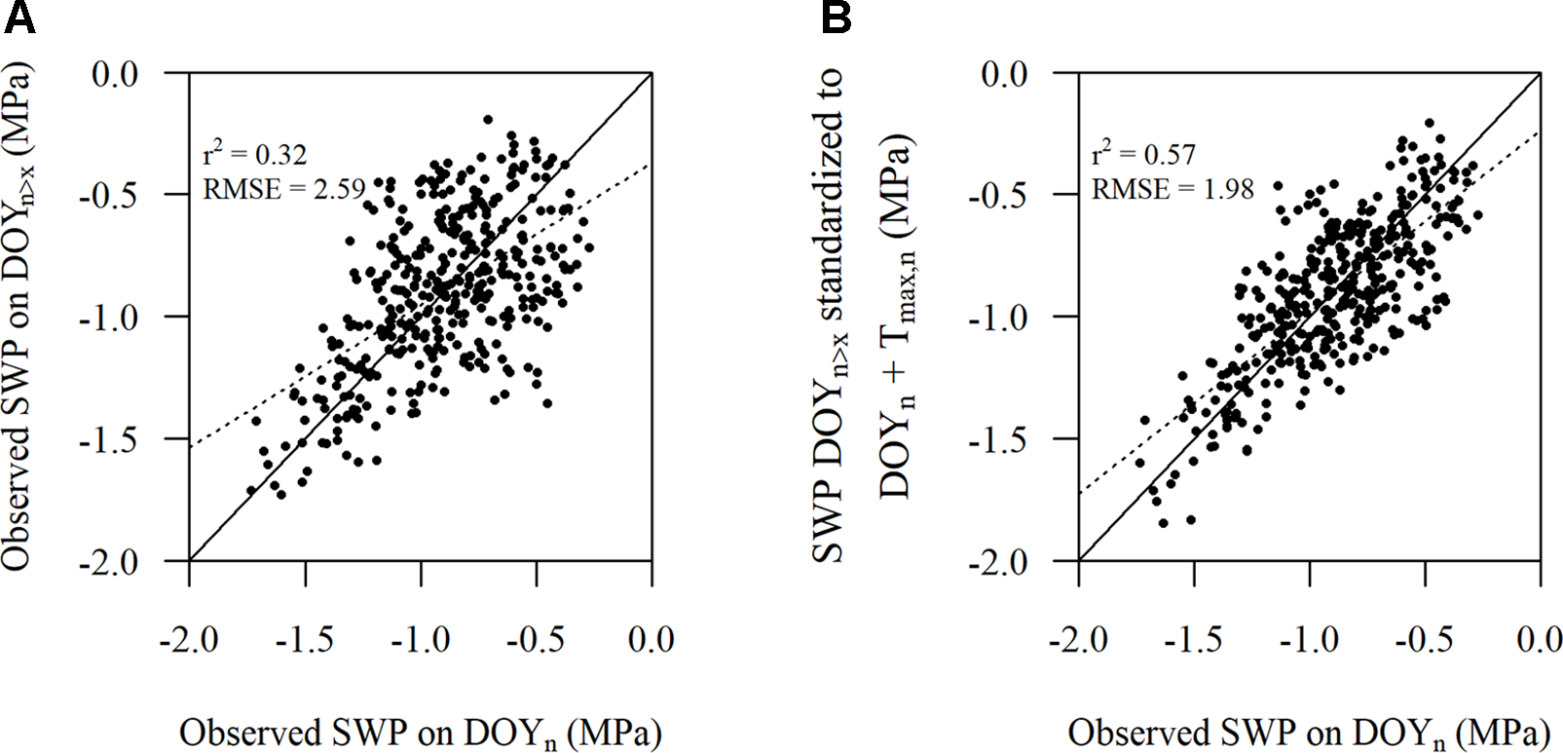
Comparison of **(A)** the observed stem water potential (SWP) on DOY_n_ versus the observed SWP on DOY_n_>x, where x is at least greater than 2 days and has a predawn leaf water potential almost equal to the observed SWP on DOY_n_ and **(B)** the observed SWP on DOY_n_ versus the observed SWP at that later date standardized according to Eqn. 2. The solid line represents the 1:1 line and the dotted line represents the linear regression (n = 385).

There were 385 observed SWP values within the plots that did have PLWP values that were almost the same or did not differ more than 0.005 MPa. The number of days between these observations could range from 1 week up to 11 weeks. Eqn. 2 showed to improve r^2^ and reduce RMSE (**Figure 5B**), compared to unstandardized SWP values (**Figure 5A**). The improvement in RMSE between the observed SWP and standardized SWP by Eqn. 2 was almost 24%.

## Discussion

The effect of soil water availability (PLWP), climatic conditions (T_max_), and seasonality (DOY) explained 80% of the variance in SWP (model 5), where 66% of the variability in SWP could be explained by PLWP through an exponential term. In line with our results, Abrisqueta et al. (2015) found that for peach 61% of the variability in SWP could be explained by soil water availability. In their modeling exercise they were able to explain 72% of the total variability in SWP by also including VPD and seasonality (as accumulated growing-degree-hours).

The relationships between SWP and T_max_ and VPD_max_ were best captured by power functions. In line with our results, previous research has already shown that VPD was nonlinearly related to SWP in both grapevines (Gálvez et al., 2014) and apple trees (De Swaef et al., 2009). The same nonlinearity has been shown for T_max_ and SWP in lemon trees (Ortuño et al., 2006; Ortuño et al., 2009), although not yet in grapevine. It is surprising that T_max_ explained more of the variability in SWP than did ET_0_ and VPD_max_, since the latter two are estimates of evaporative demand. Santesteban et al. (2011) found similar results, where SWP of Tempranillo was better estimated by T than by VPD. They reported a correlation between T and VPD of 0.82 (versus 0.83 in this study) and justified their result by the fact that the range of VPD values was quite narrow in their study. In the maritime climate of Bordeaux maximum VPD is reached mid-July, after which it decreases. In this study, ET_0_ was shown not to be a good estimator, possibly because of a large effect of global radiation early in the season on ET_0_ values.

The use of instantaneous climatic data did not appear to produce better modeling results than daily climatic data (ΔAIC > 4.5). Strong correlations existed between instantaneous and daily maximum values of T, VPD, and ET_0_ (r > 0.94). The average variability in air temperature for the part of the days wherein SWP measurements were performed equaled only 1.06°C. This degree of variability might have been too small to detect any change in SWP. Additionally, the increase in precision might have been offset by within-vine variability of SWP (0.18 MPa; **Table S2**). From a practical point of view, the advantage of T_max_ is that it is easy to acquire from basic field weather stations. Moreover, these are rarely equipped with sensors to measure climatic variables such as ET_0_ or relative humidity, which makes T_max_ an ideal variable for SWP modeling.

Temperature and rainfall data were collected very close to the experimental blocks in Saint-Émilion in 2015 (less than 500 m). Hence, climate data can be considered as highly accurate for this dataset. In 2018, the distance from the blocks to the weather station ranged from 0,710 to 1,460 km. This distance has very little impact on the accuracy of the temperature data, because the region is flat and temperatures are not very variable over short distances (Van Leeuwen et al., 2014; Bois et al., 2018). Rainfall can, however, vary over short distances and there can potentially be some discrepancy between rainfall data collected in the weather station and the actual amount of rainfall received on the block where the SWP were measured. Although grapevine cultivars are known to respond very distinctly to water deficits (Chaves et al., 2010; Santesteban et al., 2019), model 2 was only marginally improved when fitted separately for Cabernet franc, Cabernet-Sauvignon, and Merlot. It has been reported that these three cultivars show anisohydric behavior. Further investigation is needed to test if the models proposed here do apply with the same accuracy to cultivars with (near-)isohydric behavior.

In addition to PLWP and T_max_, seasonality (DOY) was found to explain a large part of the remaining variability in SWP. The seasonality in this study correlated moderately high with SWP (r = −0.64, P < 0.001) and is in line with that found by Abrisqueta et al. (2015), where seasonality was expressed as accumulated growing-degree-hours (r = −0.67, P < 0.001). Adding GDD instead of DOY improved the models, albeit marginally. Vine phenology is, however, not only determined by GDD, but also by temperature requirements of cultivars (Parker et al., 2013). It is out of the scope of this study to produce cultivar-specific models. DOY was therefore preferred to GDD. Olivo et al. (2009) demonstrated seasonal sensitivity of SWP to VPD in a 3-year field experiment in grapevine. They argued that differences between SWP in irrigation treatments at various phenological stages could be explained by an imbalance between water supply and canopy demand. However, such an imbalance caused by differential canopy demand was unlikely to be of great influence as canopy size was strictly controlled by hedging in this study. Grapevines therefore have other ways to adjust their water use to soil water availability.

Vine size is a factor potentially impacting water potentials which has not been taken into account in the models. Leaf area was measured in 2015 on the experimental blocks and varied from 1.36 to 2.30 m^2^ when fully established at the end of the season. Primary leaf area varied much less than secondary leaf area (0.67 to 0.98 and 0.53 to 1.57 m^2^, respectively). No data is available for leaf area in the experimental blocks in 2018. Vine size is likely to have an impact on SWP, which is measured during the day when vines are potentially transpiring. PLWP is measured at dawn when vines are not transpiring and should be less impacted by leaf area, because at dawn water potentials in the plant adjust with water potentials in the soil. Generally, vines with a bigger canopy also have a more developed root system. Although root to shoot ratio may vary under changing light conditions or nitrogen availability, it is rather stable for a given set of environmental conditions (Grechi et al., 2007). As long as shoot to root ratio remains unchanged, vines with bigger canopies also have access to greater water reserves and the impact of leaf area on SWP can thus be supposed to remain limited. Including vine-specific leaf area could, however, improve the models presented here and can be an area of further investigation. Vine specific leaf area is not very easy to measure (Trégoat et al., 2001) and including this parameter in the models would restrain their application in a production context.

The performance of the models developed in this study would still need to be assessed in (extremely) wet years, given that they were parameterized using data collected in two dry years. Should PLWP stay between 0 and −0.1 MPa over the course of a season, then the seasonality term in model 5 (**Table 2**) could underestimate the SWP value and result in positive SWP values. This would be the case if observed SWP is higher than −0.47 MPa at mid-September. In our dataset, a single grapevine in plot F attained a SWP of −0.42 MPa on 14 September 2018, attesting the necessity for recalibration of our model in wet years.

The standardization exercises following the proposed models allow for the comparison of SWP values obtained across dates with variable climatic conditions and different soil water availability. The added value, compared to the baseline approach, is that growers will be able to correct SWP values at levels below those of the baseline established for non-limiting water uptake conditions. This is of significant interest since a moderate water deficit is considered favorable to wine quality (Van Leeuwen et al., 2009). The advantage of this standardization over the use of PLWP is that it does not require the actual measurement of that PLWP. The PLWP measurement remains, however, useful as it represents a value close to the soil matrix potential.

The aim of this study was to provide models that separate effects of soil moisture and climatic conditions on the SWP value. Although the models explain a large part of the variability in SWP values, some of the variance remains unexplained. Part of this variability may be due to the inability of PLWP to represent plant water status when only a small part of the soil contains readily available water while transpiration demand is high (Améglio et al., 1999). Another part of the remaining variance may be explained by grapevines acclimating to water deficit by modifying hydraulic properties and gas exchange (Hochberg et al., 2017). Drought-acclimated grapevines could maintain higher gas exchange under drought, resulting in more negative SWP values compared to vines that were not previously exposed to water deficit. These two leads may be starting points to further improving the models proposed in this study.

## Conclusions

PLWP, T_max_, and DOY were main contributors to estimating SWP. Nonlinear models based on these variables were able to explain 80% of the variance in SWP for Merlot, Cabernet-Sauvignon, and Cabernet franc in field conditions. The algebraic rearrangement of the proposed models allows for standardization of SWP values to a value under predefined reference climatic conditions, in order to better reflect soil water availability. This allows for comparison of SWP values under different climatic conditions. A fine-tuned interpretation of SWP values across a wide range of water deficit would help growers to adapt management strategies to drought, both in the short term by means of irrigation, adjustment of the canopy size or vineyard floor management, or in the long term, by choosing appropriate plant material (rootstocks and cultivars) and planting densities. More research, however, is needed to better understand the effect of seasonality on the relations between climatic conditions and SWP over a wide range of PLWP values. Improvements to the model may be investigated by improving predictability of PLWP under heterogeneous soil humidity as well as accounting for acclimation to water deficit.

## Data Availability Statement

The datasets generated for this study are available on request to the corresponding author.

## Author Contributions

DP, CvL, and RT contributed conception and design of the study. BS organized the database. BS and ZD performed the statistical analysis. RT and BS wrote the first draft of the manuscript. CvL and ZD wrote sections of the manuscript. All authors contributed to manuscript revision, read and approved the submitted version.

## Funding

The study has been carried out with financial support from the French National Research Agency (ANR) in the frame of the Investments for the future Program, within the Cluster of Excellence COTE (ANR-10-LABX-45). It has not been formally reviewed by ANR. The views expressed in this publication are solely those of the authors, and ANR does not endorse any products or commercial services mentioned in this publication. This work was supported by the metaprogramme Adaptation of Agriculture and Forests to Climate Change (AAFCC) of the French National Institute for Agricultural Research (INRA), especially through the Laccave 2.21 project.

## Conflict of Interest

The authors declare that the research was conducted in the absence of any commercial or financial relationships that could be construed as a potential conflict of interest.
