# Supplementary material for: Modeling Stem Water Potential by Separating the Effects of Soil Water Availability and Climatic Conditions on Water Status in Grapevine (Vitis vinifera L.)
Source: Front Plant Sci. 2019 Nov 22;10:1485. doi: 10.3389/fpls.2019.01485 (PMC6883387; doi:10.3389/fpls.2019.01485)
Supplement: Supplementary file 1 [file Table_1.docx]

Supplementary Material

# Supplementary Data

Supplementary table 1. Dates of measurements and seasonal climatic variables throughout the course of the study.

| Date | Max Temp (°C) | Min rel. humidity (%) | Max VPD (kPa) | Global radiation (MJ/m^2^) | Daily ET_0_ (mm) | Average wind speed (m/s) |
| --- | --- | --- | --- | --- | --- | --- |
| 08/06/2015 | 30.2 | 33 | 2.87 | 28.87 | 7.40 | 3 |
| 22/06/2015 | 30.2 | 19 | 3.48 | 30.03 | 6.30 | 2 |
| 23/06/2015 | 25.5 | 29 | 2.32 | 22.18 | 5.00 | 2 |
| 29/06/2015 | 32.0 | 21 | 3.76 | 29.18 | 5.40 | 1 |
| 30/06/2015 | 38.5 | 18 | 5.58 | 28.24 | 8.00 | 3 |
| 06/07/2015 | 33.8 | 32 | 3.58 | 28.39 | 6.30 | 2 |
| 08/07/2015 | 25.1 | 36 | 2.04 | 21.04 | 5.60 | 3 |
| 23/07/2015 | 29.0 | 33 | 2.68 | 28.07 | 5.60 | 2 |
| 28/07/2015 | 27.9 | 33 | 2.52 | 14.56 | 4.30 | 2 |
| 11/08/2015 | 31.4 | 38 | 2.85 | 23.03 | 4.80 | 2 |
| 18/08/2015 | 25.6 | 37 | 2.07 | 23.33 | 4.40 | 2 |
| 20/08/2015 | 28.7 | 34 | 2.60 | 24.01 | 4.10 | 1 |
| 25/08/2015 | 28.0 | 36 | 2.42 | 22.49 | 4.60 | 2 |
| 28/08/2015 | 31.4 | 43 | 2.62 | 21.99 | 4.40 | 1 |
| 07/09/2015 | 23.8 | 29 | 2.09 | 22.10 | 4.30 | 3 |
| 09/09/2015 | 27.8 | 26 | 2.76 | 19.51 | 3.50 | 1 |
| 20/06/2018 | 32.0 | 45 | 2.61 | 28.88 | 5.51 | 2 |
| 22/06/2018 | 25.6 | 35 | 2.13 | 30.09 | 5.61 | 5 |
| 29/06/2018 | 30.6 | 39 | 2.68 | 28.79 | 5.32 | 2 |
| 30/06/2018 | 33.1 | 44 | 2.83 | 27.00 | 5.52 | 3 |
| 26/07/2018 | 33.6 | 44 | 2.91 | 25.20 | 4.78 | 1 |
| 27/07/2018 | 27.8 | 57 | 1.61 | 16.72 | 3.36 | 2 |
| 01/08/2018 | 29.1 | 49 | 2.05 | 24.82 | 4.75 | 3 |
| 02/08/2018 | 35.1 | 39 | 3.45 | 25.61 | 4.49 | 0 |
| 06/08/2018 | 36.3 | 24 | 4.59 | 25.13 | 4.26 | 0 |
| 07/08/2018 | 27.1 | 67 | 1.18 | 13.06 | 2.96 | 4 |
| 16/08/2018 | 29.7 | 48 | 2.17 | 19.65 | 3.68 | 2 |
| 17/08/2018 | 24.6 | 45 | 1.70 | 22.81 | 3.97 | 3 |
| 21/08/2018 | 31.9 | 41 | 2.79 | 22.59 | 4.25 | 2 |
| 22/08/2018 | 33.4 | 41 | 3.03 | 21.94 | 3.99 | 1 |
| 27/08/2018 | 26.6 | 66 | 1.18 | 9.74 | 1.94 | 1 |
| 28/08/2018 | 33.6 | 40 | 3.12 | 19.25 | 4.22 | 4 |
| 12/09/2018 | 31.6 | 43 | 2.65 | 17.44 | 3.16 | 2 |
| 14/09/2018 | 24.7 | 50 | 1.56 | 16.48 | 2.53 | 1 |

Supplementary table 2. SWP measured on 8 leaves on each of 5 adjacent vines located in plot A on 30 June 2018.

|  | SWP (-MPa) | | | | | |
| --- | --- | --- | --- | --- | --- | --- |
| Leaf nr. | vine 1 | vine 2 | vine 3 | vine 4 | vine 5 |  |
| 1 | 0.45 | 0.37 | 0.46 | 0.63 | 0.44 |  |
| 2 | 0.52 | 0.47 | 0.47 | 0.65 | 0.44 |  |
| 3 | 0.54 | 0.47 | 0.47 | 0.66 | 0.45 |  |
| 4 | 0.60 | 0.48 | 0.49 | 0.66 | 0.50 |  |
| 5 | 0.60 | 0.51 | 0.52 | 0.69 | 0.51 |  |
| 6 | 0.62 | 0.56 | 0.52 | 0.71 | 0.52 |  |
| 7 | 0.63 | 0.58 | 0.53 | 0.71 | 0.54 |  |
| 8 | 0.65 | 0.59 | 0.62 | 0.75 | 0.63 |  |
|  |  |  |  |  |  | Grand mean |
| Mean | 0.58 | 0.50 | 0.51 | 0.68 | 0.50 | 0.55 |
| Standard deviation | 0.07 | 0.07 | 0.05 | 0.04 | 0.06 | 0.06 |
| Absolute difference (leaf 8 - leaf 1) | 0.20 | 0.22 | 0.16 | 0.12 | 0.19 | 0.18 |
